# Longitudinal Relationship Between Bullying Victimization and Depression Among Left-Behind Children: Roles of Negative Thoughts and Self-Compassion

**DOI:** 10.3389/fpsyg.2022.852634

**Published:** 2022-03-28

**Authors:** Ru Yan, Ruibo Xie, Min Jiang, Jiayi Li, Xiuyun Lin, Wan Ding

**Affiliations:** ^1^Parent Education Research Center in Zhejiang Normal University, Key Laboratory of Intelligent Education Technology and Application of Zhejiang Province, Jinhua, China; ^2^Institute of Developmental Psychology, Beijing Normal University, Beijing, China; ^3^Beijing Key Laboratory of Applied Experimental Psychology, School of Psychology, Beijing Normal University, Beijing, China

**Keywords:** bullying victimization, depression, negative thoughts, self-compassion, left-behind children

## Abstract

**Background:**

Left-behind children (LBC) in China have aroused widespread concern in society and the academic field because they have a high risk of psychological problems. For left-behind children, depression is the most serious problem. Bullying victimization has been evidenced as one of the most common causes of children’s depression. However, less is known about its longitudinal association and the process for how bullying victimization influences depression among left-behind children. Thus, the presentation aims to explore the mechanisms underlying by considering the roles of left-behind children’s negative thoughts and self-compassion.

**Methods:**

The 3-wave longitudinal data were collected from a sample of 605 aged 8–11 from central China. We used the Olweus bully and victimization questionnaire, the children’s automatic thoughts scale, the depression scale, and the self-compassion scale.

**Results:**

Bullying victimization positively predicted the depression level of left-behind children. Negative thoughts and self-compassion mediate the relationship between bullying victimization and depression. In the mechanism of bullying victimization on depression exists gender differences among left-behind children.

**Conclusion:**

The present study suggested the association between bullying victimization and left-behind children’s depression and revealed the internal mechanism of negative thoughts and self-compassion. These findings provide a new perspective for left-behind children’s mental health education and intervention.

## Introduction

Driven by the rapid economic development, a large number of rural laborers go out to work to change their living conditions. For various reasons, they cannot take their children with them, resulting in many left-behind children. Left-behind children (LBC) refer to rural children below 18 years left at home with their grandparents or relatives when one or both of their parents migrate to an urban area for work (Su et al., 2013). There are more than 61 million LBC in China, accounting for approximately 21.9% of the total number of children in China (Li, 2021; Wang and Liu, 2021). Substantial evidence has shown that LBC has many psychological adaptation problems due to the poor living environment, parent–child separation, and lack of effective supervision and education (Weisz et al., 2006; Wen and Lin, 2012; Wu et al., 2021). Depression has become one of the most common psychological problems among LBC in rural areas (Xiao et al., 2020), which has attracted vaster attention in the public health field and educational fields. Depression is a kind of negative affective disorder caused by individuals unable to cope with stressful events in life (Thapar et al., 2012). Prior research has indicated that LBC’s depression is significantly higher than that of other children (Liu et al., 2015), and the total depression rate is as high as 26.4% (Ding et al., 2019). Depression has a significant negative impact on children’s academic performance (Hishinuma et al., 2012) and social development (Rock et al., 2014). In addition, depression in childhood may result in other mental diseases in the future (Copeland et al., 2013). Therefore, further explore the influencing factors and mechanism of LBC’s depression, which will provide effective intervention programs for LBC’s depression and have important practical significance for reducing depression and improving the mental health of LBC.

### Bullying Victimization and LBC’s Depression

Bullying victimization refers to a situation where a vulnerable person repeatedly suffers deliberate harm from one or more people and they do not harm others (Olweus, 2013). Bullying victimization is relatively common among LBC. A survey conducted by the Institute of Psychology of the Chinese Academy of Sciences released that 15% of LBC had suffered violence (Institute of Psychology, CAS, 2016). According to the interpersonal risk model, a negative interpersonal interaction environment can lead to a series of emotional problems including depression (Patterson and Capaldi, 1990). As a negative interpersonal interaction between peers, bullying victims may experience depression. When children face the negative interpersonal stress environment of bullying victimization, it will increase the child’s perceived stress (Chang and Lu, 2018; Lu and Chang, 2019), which will lead to emotional problems, such as depression. Previous studies have suggested that being bullied for a long time will cause more physical and mental problems, such as stress, fear, and anxiety (Schwartz et al., 2015; Troop-Gordon, 2017). Moreover, bullying victimization increased individual depression, and suicidal behavior occurs in severe cases (Lereya et al., 2015).

In school, LBC were more likely to be bullied by others (Chen and Chan, 2016; Zhu et al., 2020). For LBC, due to the absence of parents and lack of parent–child communication (Wang et al., 2002), they may not be able to seek help from their parents timely when they were bullied (Otake et al., 2019). They cannot effectively deal with the emotional consequences of being victims of bullying and that negative emotion may be inward, leading to depression (Cheng and Sun, 2015; Liu et al., 2015; Ding et al., 2019). Previous studies have focused on the family environment, such as parents, cross-sectional, while school environment, another important place for LBC, has received less attention. Bullying victimization is a very serious and common phenomenon in school, which deserves further investigation. Therefore, our study will use a longitudinal inquiry to examine the influence of bullying victimization on LBC’s depression.

### The Mediating Role of Negative Thoughts and Self-Compassion

The psychological mediation frameworks reveal that distal stress processes further influence their development by influencing proximal individual factors (Hatzenbuehler, 2009). Among them, distal stressors often refer to events, such as violence, and proximal factors usually refer to cognitive processes, self-regulation, etc. Negative thoughts will cause individuals to have negative perceptions and pessimistic interpretations when facing pressure (Yu et al., 2022), which is a typical bad cognitive process. For LBC, both parents leave home all year-round, parental absence contributes to reduced or absent family resources, such as a lack of support and involvement (Zhao et al., 2015). LBC faces a higher risk of being bullied (Hu et al., 2018), and they have easily produced more negative thoughts, further triggering depression. Meanwhile, bullying victimization may weaken some positive factors of children, thus increasing depression. Self-compassion is a very positive self-regulation strategy that helps reduce negative emotions, such as depression by acknowledging discomfort and self-kindness, contributing to healthy mental development (Neff, 2003). Previous studies have shown that negative thoughts and self-compassion are important factors that may mediate the association between adverse experiences and depressive symptoms (Wu et al., 2018; Hou et al., 2020). Therefore, this study will simultaneously explore the mediating role of negative thoughts and self-compassion in the relationship between bullying victimization and depression in LBC. Meaningful guidance and advice will be provided for this special group.

Negative thoughts refer to a kind of thinking flow with negative content, automatic emergence, and often associated with negative emotions (Beck and Perkins, 2001). In other words, negative thoughts are the automatic negative thoughts that constantly appear in the mind, such as “I am bad” and “No one loves me.” Beck’s cognitive theory holds that when individuals encounter stressful events, they show deep-seated dysfunctional attitudes, which will lead to negative thoughts and a negative view of themselves, society, and the future, which may easily lead to depression (Beck, 2002). Negative thoughts are often activated by negative life experiences and will directly lead to the generation and maintenance of depression. Several empirical studies have found that children bullied by others can guide the appearance of negative thoughts icons (Roth et al., 2002; Storch and Ledley, 2005). These children may generally have psychological and emotional disorders and more negative thoughts. Then it will increase their depression level (Buschmann et al., 2018). Such a challenge for LBC, the parent–child relationship is estranged, and the living conditions are weak, form their psychological sensitive, fragile, stressful events are likely to increase their negative thoughts, which may lead to depression (Wu et al., 2017). Previous research on negative thoughts mainly focused on ordinary groups. No studies have investigated the role of negative thoughts in the relationship between bullying victimization and LBC’s depression.

Self-compassion is about fully accepting oneself while in pain and as a positive self-attitude and self-regulation. It describes individuals who do not avoid their pain and failure, feel open and tolerant, and give an unbiased understanding (Neff, 2003). Neff (2003) conceptualized self-compassion in three primary features: self-kindness, common humanity, and mindfulness. The core of self-compassion is thought to be the ability to treat oneself kindly after something has happened (Allen and Leary, 2010). Empirical research has indicated that bullying victimization may deprive children of their ability to care for themselves (Doyle and Sullivan, 2017). It can be further interpreted that when LBC bullying victimization, they usually choose to avoid pain, suppress emotions, and self-denial, which may make a lower level of self-compassion (Neff and Mcgehee, 2010). Some studies have found that self-compassion neutralizes the harmful effects of self-critical thoughts on depression and prevents individuals from suicidal behavior (Neff and Mcgehee, 2010; Raes, 2011). Individuals with self-compassion pay attention to the recent experience in a balanced and calm manner (Germer and Neff, 2013), relieve their fears, and reduce depression (Collett et al., 2016). A recent study has shown that self-compassion plays an intermediary role in the relationship between peer victimization and depression symptoms in adolescents (Yaghoubi et al., 2021). Few studies have been investigated on the part of self-compassion in LBC. However, because of the particularity of LBC, we should also attach importance to the positive factors of LBC. Therefore, this study will explore the role of self-compassion in the relationship between bullying victimization and LBC’s depression.

### Gender Differences

Several researchers have reported significant gender differences in the development level of bullying victimization and depression among adolescents. Gender differences in depression can be seen as serious health disparities, in which females are more prone to depression than males (Nolen-Hoeksema and Hilt, 2009; Hyde and Mezulis, 2020). The proportion of males who suffered from bullying was markedly higher than that of females (Fleming and Jacobsen, 2010; Atik and Güneri, 2013; Huang et al., 2013). But bullying victimization has a stronger predictive effect on depression in females than in males (Klomek et al., 2007). Moreover, previous studies have found that females and males have different gender roles, so there are differences in self-compassion, among which females have slightly lower levels of self-compassion than males (Yarnell et al., 2019). However, few studies have further revealed whether there are gender differences in the relationship between bullying victimization and depression and the potential mechanisms among LBC.

### The Present Study

In summary, based on the interpersonal risk model and psychological mediation framework, this study using a longitudinal survey design aimed to investigate the longitudinal association between bullying victimization and depression among LBC, and to further examine the mediating role of negative thoughts and self-compassion. Four research hypotheses will be proposed by us as follows.

*Hypothesis 1*: bullying victimization significantly predicts LBC’s depression.

*Hypothesis 2*: negative thoughts play a mediating role in the relationship between bullying victimization and LBC’s depression.

*Hypothesis 3*: self-compassion mediates the relationship between bullying victimization and LBC’s depression.

*Hypothesis 4*: there are gender differences in the mechanism of bullying victimization and LBC’s depression.

A previous study has found that age affects depression (Liu et al., 2015), so this study will control the age.

## Materials and Methods

### Participants

We recruited 605 LBC from central China to complete the first survey (T1). For the second time (T2), 573 LBC (94.71%) participated and completed. For the third time (T3), 529 LBC (87.44%) completed the survey. The age of participants ranged from 8 to 11 years, and their average age was 9.56 years (*SD* = 0.79). Among them, the average age of females was 9.49 (*SD* = 0.73); the average age of males was 9.60 (*SD* = 0.82).

Before implementing the research measures, the Zhejiang Normal University Institutional Review Board approved this follow-up study. This study was conducted with the consent of the school and the child’s guardian. The principal investigators were professionally trained psychology graduate students. They explained the specific meaning of each item and guided the students to answer it independently with the help of the teacher in charge. The students were told that none of their responses would be revealed to anyone and that they could stop participating at any time without penalty. All participants completed a pen-and-paper questionnaire in Chinese within 1 h.

### Measures

#### Bullying and Victimization

This study used from the Olweus Bully and Victimization Questionnaire. The questionnaire was revised by Zhang and Wu (1999), including three dimensions: physical bullying, verbal bullying, and relational bullying. The scale has six items. Each item is rated on a five-point Likert scale ranging from 0 (never happened this semester) to 4 (several times a week). The higher total score represents a higher degree of bullying or being bullied. In this study, Cronbach’s alphas at time 1 was 0.94.

#### Negative Thoughts

Children’s Automatic Thoughts Scale (CATS) was developed by Schniering and Rapee (2002). The scale includes 40 items. Four dimensions are a personal failure, physical threat, interpersonal threat, hostility. Each dimension contains ten items. Each item is rated on a five-point Likert scale ranging from 0 to 4. A higher score indicates more negative thoughts. In this study, at time 2, Cronbach’s alphas was 0.98.

#### Self-Compassion

Gong et al. (2014) revised Neff’s self-compassion scale to get the self-compassion questionnaire. The scale has 12 items, including three dimensions of self-kindness, common humanity, and mindfulness. Each item is rated on a 5-point scale (1 = rarely to 5 = almost always). In this study, at time 2, Cronbach’s alphas was 0.82.

#### Depression

Children Depression Scale (CDS-DC) was compiled by Fendrich et al. (1990) and revised by William Li et al. (2010). The scale has 20 items and deals with the six main symptoms of depression. All items are rated on a four-point Likert scale ranging from 0 (never) to 3 (always). The higher the score reflects, the more serious the degree of depression. In this study, at time 2-time 3, Cronbach’s alphas were 0.89, 0.86.

### Analysis Plan

SPSS 21.0 and Mplus 8.3 were employed to analyze the data. Missing data were handled using Expectation–Maximization (EM). A chi-square test, comparative fit index (CFI), Tucker–Lewis index (TLI), root mean square error of approximation (RMSEA), and standardized root mean residual (SRMR) were reported to examine the model fit. The Bootstrap method is used to test the mediation effect.

## Results

### Descriptive Statistics and Correlations

After centralizing the data, the means, standard deviations, and correlations for all variables are shown in Table 1. In the female group, the correlation analysis showed that victimization at time 1, negative thoughts at time 2, and depression at time 3 were positively correlated (*p* < 0.001). Self-compassion at time 2 was negatively correlated with at time 1 victimization, and negative thoughts at time 2 (*p* < 0.001, *p* < 0.05). In the male group, the correlation analysis showed that victimization at time 1, depression at time 3, and negative thoughts at time 2 were positively correlated (*p* < 0.001); self-compassion at time 2 was significantly negatively correlated with victimization at time 1, negative thoughts at time 2, and depression at time 3 (*p* < 0.001, *p* < 0.01).

**Table 1:** Means (*M*), standard deviations (*SD*), and bivariate correlations of key study variables.

	Females *M ± SD*	Males *M ± SD*	1	2	3	4	5
1. T1 Victimization	0.83 ± 1.12	1.04 ± 1.14	–	0.33^***^	−0.17^**^	0.26^***^	0.25^***^
2. T2 Negative thoughts	0.82 ± 0.89	1.07 ± 1.00	0.33^***^	–	−0.14^**^	0.38^***^	0.35^***^
3. T2 Self-Compassion	3.21 ± 0.38	3.22 ± 0.41	−0.17^*^	−0.27^***^	–	−0.27^***^	−0.23^***^
4. T2 Depression	1.15 ± 0.50	1.19 ± 0.50	0.33^***^	0.38^***^	−0.29^***^	–	0.24^***^
5. T3 Depression	0.94 ± 0.45	1.00 ± 0.47	0.28^***^	0.34^***^	−0.11	0.34^***^	–

*Correlations in top of diagonal are for males, in the bottom for females. T1*, *Time 1; T2*, Time 2; T3, Time 3. ^***^p < 0.001, ^**^p < 0.01, ^*^p < 0.05.

### Structural Model

First of all, we analyze the data of all LBC (*n* = 605). Victimization at time 1 as the independent variable, negative thoughts and self-compassion at time 2 as the mediating variables, and depression at time 3 as the dependent variable, the mediating model, were tested. The results showed that the full measurement model was satisfactory: *χ*^2^(4) = 7.88, CFI = 0.99, TLI = 0.95, RMSEA = 0.04, SRMR = 0.02.

**Figure 1:**
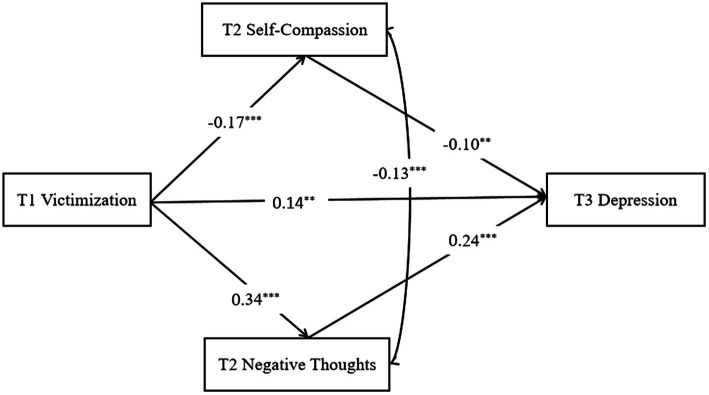
Serial mediation model among all LBC. All the coefficients are standardized estimates. For simplicity of the model, the control variables are not shown in the figure. We controlled for age, gender, and T2 depression.

Through the analysis of each path in the model, in Figure 1, it is found that victimization at time 1 can positively predict negative thoughts at time 2 (*β* = 0.34, *p* < 0.001) and depression at time 3 (*β* = 0.14, *p* < 0.01); negative thoughts at time 2 significantly predicts depression at time 3 (*β* = 0.24, *p* < 0.001); victimization at time 1 negatively predicts self-compassion at time 2 (*β* = −0.17, *p* < 0.001); and self-compassion at time 2 significantly negatively predicted depression at time 3 (*β* = − 0.10, *p* < 0.01).

The medium effect test was carried out using the bootstrap estimation procedure (with 1,000 bootstrapping samples), as shown in Table 2. The 95% confidence intervals of the mediating effects of negative thoughts at time 2 and self-compassion at time 2 on victimization at time 1 and depression at time 3 were [0.05, 0.12] and [0.004, 0.04], which did not contain 0, indicating that the mediating effect was significant.

**Table 2:** Standardized path coefficients.

Model pathways	Effects	95%CI	
		Lower	Upper
T1 Victimization → T2 Negative thoughts → T3 Depression	0.08	0.05	0.12
T1 Victimization → T2 Self-Compassion → T3 Depression	0.02	0.004	0.04

**Figure 2:**
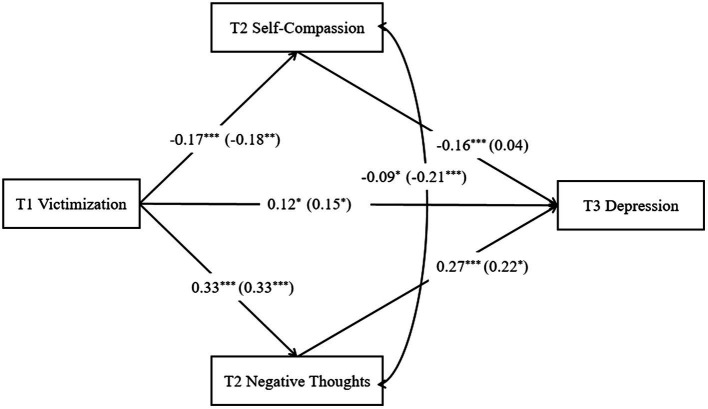
Serial mediation model male (female). All the coefficients are standardized estimates. For simplicity of the model, the control variables are not shown in the figure. We controlled for age and T2 depression.

Then, the study further analyzed the data of female and male, as shown in Figure 2. In female group (*n* = 210), the moderated mediation model showed an acceptable model fit with *χ*^2^(4) = 1.39, CFI = 0.99, TLI = 0.96, RMSEA = 0.04, SRMR = 0.02. Through the analysis of each path in the female model, it is shown that victimization at time 1 positively predicts negative thoughts at time 2 and depression at time 3 (*β* = 0.33, *p* < 0.001; *β* = 0.15, *p* < 0.05); negative thoughts at time 2 significantly positively predicts depression at time 3 (*β* = 0.22, *p* < 0.05); victimization at time 1 can negatively predict self-compassion at time 2 (*β* = −0.18, *p* < 0.01); self-compassion at time 2 did not predict depression at time 3.

In male group (*n* = 395), it has showed an adequate overall model fit, *χ*^2^ (4) = 1.415, CFI = 1, TLI = 1.04, RMSEA = 0.00, SRMR = 0.01. Through the analysis of each path in the male model, it is found that victimization at time 1 positively predicts negative thoughts at time 2 and depression at time 3 (*β* = 0.33, *p* < 0.001; *β* = 0.12, *p* < 0.05); negative thoughts at time 2 significantly positively predicted depression at time 3 (*β* = 0.27, *p* < 0.001); victimization at time 1 can negatively predict self-compassion at time 2 (*β* = −0.17, *p* < 0.001); self-compassion at time 2 significantly predicts depression at time 3 (*β* = −0.16, *p* < 0.001).

As shown in Tables 3, 4, using bootstrap estimation procedure (with 1,000 bootstrapping samples) to test the mediating effects. In the female group, the 95% confidence interval of the mediating effect of negative thoughts at time 2 between victimization at time 1 and depression at time 3 was [0.01, 0.17], excluding 0, the mediating impact was significant. The 95% confidence interval of self-compassion at time 2 mediating effect was [−0.04, 0.01], including 0, indicating that the mediating effect was not significant.

**Table 3:** Standardized path coefficients (Female).

Model pathways	Effects	95%CI	
		Lower	Upper
T1 Victimization → T2 Negative thoughts → T3 Depression	0.07	0.01	0.17
T1 Victimization → T2 Self-Compassion → T3 Depression	−0.01	−0.04	0.01

**Table 4:** Standardized path coefficients (Male).

Model pathways	Effects	95%CI	
		Lower	Upper
T1 Victimization → T2 Negative thoughts → T3 Depression	0.09	0.05	0.14
T1 Victimization → T2 Self-Compassion → T3 Depression	0.03	0.01	0.05

In the male group, the 95% confidence intervals of the mediating effects of negative thoughts at time 2 and self-compassion at time 2 on victimization at time 1 and depression at time 3 were [0.05, 0.14] and [0.01, 0.05], which did not contain 0, indicating that the mediating effects were significant.

## Discussion

This study examined the longitudinal relationship of bullying victimization to depression in LBC. The results found that bullying victimization significantly affects the depression of LBC, and negative thoughts and self-compassion play a mediating role. Moreover, we found gender differences in the potential mechanism of bullying victimization and LBC’s depression. Our study uncovered the relationship and underlying mechanism between bullying victimization and depression in LBC, which has contributed to our understanding of its mechanism and provides a new perspective for the intervention of LBC’s depression.

### Bullying Victimization and LBC’s Depression

This study has shown that LBC who has the experience of bullying victimization will deliver a higher level of depression, supporting the first hypothesis. The results of this study supported the interpersonal risk model (Patterson and Capaldi, 1990) and verified the applicability of the model to rural children in China. Bullying victimization can make LBC more prone to depression and we summarize the following three main reasons. Firstly, LBC are vulnerable to discrimination and bullying from people around them (Chen and Chan, 2016). They are often rejected and isolated by peers, with constant down in spirits and loss of interest in social contact, which further alienation from peers may increase depression (Kim et al., 2005; Reijntjes et al., 2010). Secondly, generally, when the LBC are bullying victimization, the self-awareness and self-evaluation are lower than others (Prinstein et al., 2001). LBC will perceive themselves as unable to cope with the stressful events effectively, and they will not try to deal with the bullying. This negative interpersonal environment reduces LBC to adopt correct coping strategies (Prinstein et al., 2009), thereby making them vulnerable to mental health problems. In addition, bullying victimization makes LBC’s interpretation of negative events becomes subjectivity. They will think that the bullying is stable and unchangeable and feel more helpless and desperate (Siyahhan et al., 2012), therefore more prone to depression.

### The Mediating Role of Negative Thoughts and Self-Compassion

The results of this study indicate that bullying victimization increased LBC’s depression through negative thoughts and weaken LBC’s depression through self-compassion, which supports the second and third hypotheses. Meanwhile, bullying victimization, as distal pressure, affects depression levels through negative thoughts and self-compassion, which further supports the psychological mediation framework and provides a new perspective for the intervention of LBC’s depression.

Our study found that bullying victimization further increased LBC’s depression through negative thoughts. The cognitive model of depression theorized that negative experiences, such as bullying victimization, might foster negative reviews (as a form of cognitive vulnerability) due to a person’s information processing biases that lead to the appearance of depressive symptoms (Hou et al., 2020). When LBC are bullied, their normal cognitive system will collapse, and they cannot think rationally. At this time, negative thoughts are easily activated (Lu et al., 2019). Under the influence of bullying, they rationalize their injuries, increase the sense of unworthiness. Both females and males tend to use negative thoughts, evaluate themselves and others with negative views, and lead to negative results, such as depression. Therefore, after being bullied, LBC will further lead to depression through negative thoughts.

Self-compassion also plays a mediating role in the relationship between bullying victimization and LBC’s depression. It is consistent with the previous research results in non-left behind groups (Yaghoubi et al., 2021). Self-compassion appears to be an important source as a road to happiness, which positively affects the bullied children (Neff, 2011). Bullying victimization is one of the sources of stress for LBC, increasing self-blame and self-emotional dysregulation and weakening their self-compassion (Zhang et al., 2019). However, LBC with self-compassion can treat themselves well when they are being bullied. They are more likely to be kinder to themselves and understand their experiences (Allen and Leary, 2010). Then clearly perceive the current situation in a balanced way, and prevent the development of depressive symptoms.

### Gender Differences

We also found that bullying victimization can indirectly affect LBC’s depression through self-compassion in the male group, but not in the female group. This is the first time that gender differences have been found in the relationship and underlying mechanisms of bullying victimization on depression. This may be caused by females’ self-compassion levels are slightly lower than males’ (Yarnell et al., 2019). Female socialization experiences emphasize self-sacrifice to meet the needs of others, and females are more hypercritical and critical of themselves, which may lead to lower levels of self-compassion (DeVore, 2013; Sun et al., 2016). When females face the negative interpersonal relationship of bullying victimization, they may increase their susceptibility to negative consequences (Carbone-Lopez et al., 2010), and even blame and criticize themselves, and cannot further suppress depression through self-compassion. Compared to females, males have slightly higher levels of self-compassion and males attach importance to self-assertion and independence, and males may be more willing to take their own needs seriously and sympathize with themselves in times of crisis (Yarnell et al., 2019). Males tend to respond positively to negative stressful experiences, such as bullying victimization, and they will reduce the negative development through slightly higher levels of self-compassion and self-kindness (Zhang et al., 2019; Liu et al., 2020). Therefore, after bullying victimization weakens self-compassion, males can still further reduce depression levels through self-compassion.

### Limitations and Implications

This study uses a tracking design method to explore the mechanism of the impact of bullying victimization on LBC depression and examines the applicability of the interpersonal risk model and psychological mediation framework in LBC. In addition, our research results will provide important practical enlightenment for parents, schools, teachers, and psychological educators. First of all, we should prevent school bullying in time and provide timely psychological counseling for LBC who are victims. Then, we should actively cultivate students’ self-compassion ability, especially the self-compassion ability of girls. Finally, we can reduce the negative thoughts of LBC through various pieces of training and encourage positive thinking.

Our study also has some limitations. First, we mainly use children’s self-reports, considering that children may not reach cognitive maturity (Icenogle et al., 2019), and in the future, we will also consider information provided by more informants, such as parents, teachers, and peers. Second, our sample size may not be enough, and more subjects will be recruited in multiple regions in the future. Finally, this study was only tested in Chinese culture, and in the future, we will seek opportunities to collaborate with scholars in other cultures to cautiously generalize our findings to LBC in other countries.

## Data Availability Statement

The raw data supporting the conclusions of this article will be made available by the authors, without undue reservation.

## Ethics Statement

The studies involving human participants were reviewed and approved by Research Ethics Committee of Zhejiang Normal University. Written informed consent to participate in this study was provided by the participants’ legal guardian/next of kin.

## Author Contributions

RY, RX, WD, and XL conceived and designed the experiments. RY performed the experiments and analyzed the data and drafted the manuscript. RY, RX, WD, JL, MJ, and XL revised the manuscript. All authors contributed to the article and approved the submitted version.

## Funding

This research was funded by the Philosophy and Social Science Planning Project of Zhejiang Province, China (No. 22NDQN212YB), the Major Project of Humanities and Social Sciences in universities of Zhejiang Province, China (No. 2021QN064), and the Education Sciences Planning Project of Zhejiang Province (No. 2022SCG377).

## Conflict of Interest

The authors declare that the research was conducted in the absence of any commercial or financial relationships that could be construed as a potential conflict of interest.

## Publisher’s Note

All claims expressed in this article are solely those of the authors and do not necessarily represent those of their affiliated organizations, or those of the publisher, the editors and the reviewers. Any product that may be evaluated in this article, or claim that may be made by its manufacturer, is not guaranteed or endorsed by the publisher.
